# *MTHFR* gene polymorphisms in hypothyroidism and hyperthyroidism among Jordanian females

**DOI:** 10.20945/2359-3997000000133

**Published:** 2019-04-26

**Authors:** Diala W. Abu-Hassan, Abdullah N. Alhouri, Nadera A. Altork, Zakaria W. Shkoukani, Tamer Salhab Altamimi, Omar M. Alqaisi, Baha Mustafa

**Affiliations:** 1 Department of Physiology and Biochemistry School of Medicine University of Jordan Amman Jordan Department of Physiology and Biochemistry, School of Medicine, University of Jordan, Amman, Jordan; 2 School of Medicine University of Jordan Amman Jordan School of Medicine, University of Jordan, Amman, Jordan

**Keywords:** deficiency, folic acid, metabolism, thyroid, thyroxine

## Abstract

**Objective:**

Methylenetetrahydrofolate reductase (MTHFR) is involved in DNA methylation that is associated with autoimmune pathology. We investigated the association between *MTHFR* genetic polymorphisms at g.677C>T and g.1298A>C and their haplotypes, and the risk of thyroid dysfunction among Jordanian females.

**Subjects and methods:**

A case-control study involving 98 hypothyroidism cases, 66 hyperthyroidism cases and 100 controls was conducted. Polymerase chain reaction/restriction fragment length polymorphism technique was performed to determine genotypes. Statistical analysis using SPSS software was performed.

**Results:**

Genetic analysis showed a significant difference in genotype frequency of g.1298A>C between cases, and controls [hypothyroidism: AA (45.9%), AC (37.8%), CC (16.3%); hyperthyroidism: AA (9.1%), AC (69.7%), CC (21.2%); controls: AA (37.8%), AC (29.6%), CC (32.7%); CC_hypo_ vs. AA_hypo_: 2.55, 95% CI: (1.18-5.52); OR at least on C_hypo_: 1.79, 95% CI: (1.07-2.99)]; CC_hyper_ vs. AA_hyper_: 4.01, 95% CI: (1.79-9.01); OR at least on C_hyper_: 0.18, 95% CI: (0.07-0.48)]. There was no significant difference in genotype frequency of g.677C>T between cases and controls [hypothyroidism: CC (50.0%), CT (32.7%), TT (17.3%); hyperthyroidism: CC (77.3%), CT (15.2%), TT (7.6%); controls: CC (55.6%), CT (32.3%), TT (12.1%)]. There was a significant difference of *MTHFR* haplotypes among hypothyroidism cases and controls. TA and CC had a lower hypothyroidism risk whereas; TC showed a higher risk.

**Conclusions:**

g.1298A>C genetic polymorphism of *MTHFR* may modulate the risk of thyroid disease. CC, TA, and TC haplotypes affect the risk of hypothyroidism. Larger samples should be included in the future to verify the role of *MTHFR* polymorphisms in thyroid diseases.

## INTRODUCTION

Hypothyroidism is a common health problem with a worldwide annual incidence of 1.5 cases per 1,000 individuals (1,2). It occurs more often in females, with an incidence around 10-15 times higher than in males (1,2). In the United States, it has been estimated to occur in 3.5 per 1,000 women and 0.8 per 1,000 men yearly (3,4). In Jordan, the overall prevalence of thyroid disease is 12.5% (5). The worldwide prevalence of congenital hypothyroidism is 1:4,000, while in Jordan it is 1:1,719 (6). Hypothyroidism during pregnancy has been associated with gestational diabetes, premature deliveries, offspring with low intelligence, risk of peripartum death, and a higher risk of spinal cord malformations (e.g., spina bifida) and Down’s syndrome (7-9). Since the majority of those affected by Hashimoto’s auto-immune thyroiditis are females of child-bearing age, they are more inclined to experience these complications.

DNA methylation has been shown to influence gene expression in several studies (10). As an epigenetic change, DNA methylation regulates several biological events, including embryonic development, transcriptional regulation, chromatin modification, X-chromosome inactivation, and genomic imprinting (11). Changes in DNA methylation patterns have been correlated with tumorigenesis and autoimmune disease development (12).

The methylenetetrahydrofolate reductase (MTHFR) enzyme catalyzes the conversion of 5, 10-methylenetetrahydrofolate to 5-methyltetrahydrofolate. As the primary circulatory form of folate, 5-methyltetrahydrofolate is utilized to supply methyl groups in several methylation reactions. MTHFR contributes to the hypermethylation of genomic DNA (13,14). The resulting DNA hypermethylation may affect genes that influence the risk of autoimmune thyroid diseases (AITD), leading to pathological changes in thyroid gland function. The two most common genetic polymorphisms of the *MTHFR* gene worldwide are the g.677C>T and g.1298A>C variants, with the g.677C>T variant being present in 24% of the healthy Jordanian population (15). In genetically isolated groups in Jordan, such as Chechens and Circassians, the prevalence of the g.677C>T polymorphism is 27.5% and 50%, respectively (16). The *MTHFR* +677C/T polymorphism (rs1801133) results in an alanine (C)-to-valine (T) substitution and reduces the enzymatic activity of MTHFR (17,18). The *MTHFR* +1298A/C polymorphism (rs1801131) results in a glutamic acid (A)-to-alanine (C) substitution leading to a significant decrease of MTHFR enzyme activity in CC genotype individuals (19). Genetic variation in this gene affects the susceptibility to many types of cancer, including thyroid cancer (20-24).

In this case-control study, we investigated the relationship between the common genetic polymorphisms of *MTHFR*, g.677C>T, and g.1298A>C and their effect on susceptibility to thyroid dysfunction among Jordanian females.

## SUBJECTS AND METHODS

### Study population

**Figure 1:**
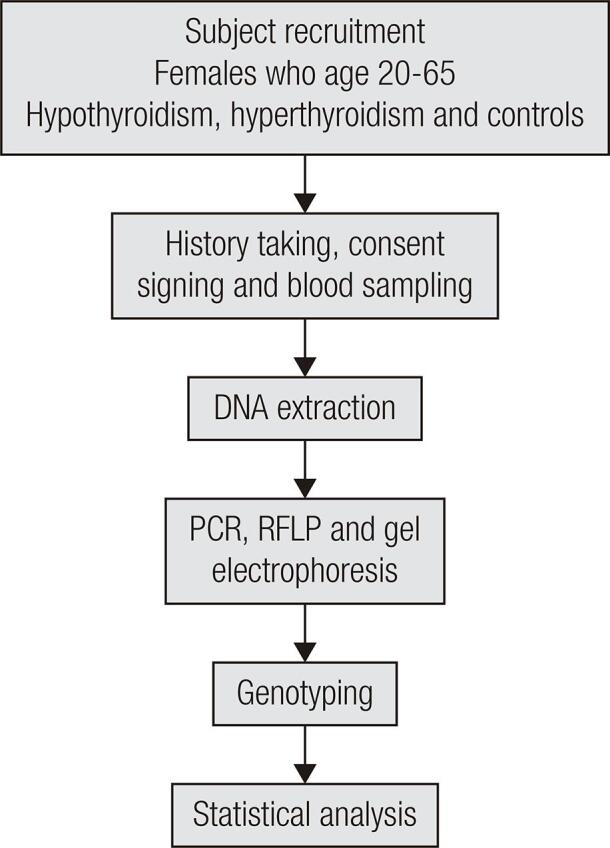
Experimental scheme.

A total of 264 female patients attending the National Center for Diabetes, Endocrinology and Genetics (NCDEG) in Amman, Jordan were recruited in this case-control study. Ninety-eight hypothyroidism patients, 66 hyperthyroidism patients, and 100 age-matched healthy women were involved (Figure 1). Hyperthyroidism patients were involved in the study for comparison. The study was approved by the institutional review board (IRB) of the NCDEG in compliance with the ethical standards of the responsible committee on human experimentation (institutional and national) and with the Declaration of Helsinki. Blood withdrawal from each subject was performed with their permission following an explanation of the purpose of the study. Each participant signed a consent form before giving a sample. The authors declared no potential conflicts of interest to study participants.

### *MTHFR* genotyping

Four milliliters of venous blood were collected from patients and healthy subjects in K3EDTA coated tubes. DNA extraction was performed the same day using QIAGEN Puregene Blood Core Kit B (QIAGEN Sciences, Maryland, USA) according to manufacturer’s instructions. The g.677C>T region (rs1801133) was amplified using the forward primer TGAAGGAGAAGGTGTCTGCGGGA (NT_021937.19: 7,861,134 to 7,861,112) and the reverse primer AGGACGGTGCGGTGAGAGTG (NT_021937.19: 7,860,937 to 7,860,956) yielding a 198-bp band. For the g.1298A>C polymorphism (rs1801131), the forward primer CAAGGAGGAGCTGCTGAAGA (NT_021937.19: 7,859,255 to 7,859,236) and the reverse primer CCACTCCAGCATCACTCACT (NT_021937.19: 7,859,128 to 7,859,147) were used, yielding a 128-bp band. The polymerase chain reaction (PCR) conditions for both polymorphisms were: 8 minutes of initial denaturation at 95ºC followed by 40 cycles of 95ºC for 60 seconds, 63ºC for 60 seconds, and 72ºC for 60 seconds, with a final extension at 72ºC for 7 minutes (Bio-Rad, C1000 Thermal cycler™, USA). After PCR was completed, the product of g.677C>T was digested with HinfI restriction enzyme. The resulting fragments were separated by 3% agarose gel electrophoresis and then visualized using RedSafe™ (iNtRON BIOTECHNOLOGY, Korea) staining (Figure 1). The digestion fragment sizes for the g.677C>T genotypes were: 198-bp bands for CC, 175, 23-bp bands for TT, and 198, 175, and 23-bp bands for CT. After PCR was completed, the products of g.1298A>C were digested with MboII restriction enzyme. The resulting fragments were separated by 3% agarose gel electrophoresis and then visualized using RedSafe™ (iNtRON BIOTECHNOLOGY, Korea) staining. The digestion fragment sizes for the g.1298A>C genotypes were: 72 and 28-bp bands for AA, 100 and 28-bp bands for CC, and 100, 72 and 28-bp bands for AC. Polymerase chain reaction-restriction fragment length polymorphism (PCR-RFLP) findings were validated by running a negative control containing all PCR components except the DNA template in every PCR run, in addition to repeating around 23% of all samples by a different lab personnel.

### Statistical analysis

Data was coded and entered into SPSS software version 16 (Chicago, IL). Data was expressed as mean ± standard deviation (SD) or as counts (%). The presence of a statistical correlation between categorical variables was evaluated by a Chi-square test. The age difference between cases and controls was evaluated by an independent student t-test. Odds ratios (OR) and 95% confidence intervals (95% CI) were assessed for measuring the association between *MTHFR* genotypes/alleles/diplotypes/ haplotypes and thyroid disorders. A *p* value < 0.05 was considered statistically significant in all analyses performed in this study. The Hardy-Weinberg equilibrium was applied to assess genotypes and allele frequencies.

### Haplotype analysis

The interaction between genetic polymorphisms at the two loci was assessed by evaluating the combined genotypes’ effects and haplotype analysis. We analyzed the haplotype frequencies of the two single nucleotide polymorphisms, SNPs, g.677C>T, and g.1298A>C for hypothyroidism and hyperthyroidism cases and compared them with those of the controls. Haplotype frequencies were calculated using Multiallelic Interallelic Disequilibrium Analysis Software (MIDAS; University of Southampton, Highfield, Southampton, UK) and linkage disequilibrium was represented by D prime (D’).

## RESULTS

A total of 264 female subjects (98 hypothyroidism patients, 66 hyperthyroidism patients, and 100 age-matched controls) were included in this study. The mean age for patients and control groups was 47.6 ± 10.0 years. No significant age differences were observed between the three arms (*p* = 0.35). Table 1 presents the distribution of the study subjects according to thyroid status in several age groups. The distribution of *MTHFR* g.677C>T and g.1298A>C genotypes is shown in Table 2. There were statistically significant differences in the genotype frequency of *MTHFR* g.1298A>C polymorphism between hypothyroidism patients and the controls. The frequency of the *MTHFR* C allele was significantly lower in hypothyroidism and hyperthyroidism patients in comparison with healthy controls. Subjects with AC and CC (g.1298A>C) genotypes had significantly lower risks of hypothyroidism than those with AA (g.1298A>C) (*p* = 0.019 and 0.017, respectively; Table 2). The C (g.1298A>C) allele is less frequent in hyperthyroidism cases when compared to controls (Table 2). The CC (g.1298A>C) genotype is less frequent in hyperthyroidism patients than in controls (Table 2). The T allele was significantly less frequent in hyperthyroidism patients compared to controls (Table 2). No other significant differences were found in the g.677C>T SNP between hypothyroidism cases, hyperthyroidism cases, and healthy controls (Table 2).

**Table 1:** Age characteristics of cases and controls

Condition		Cases	Controls	
				*p*
		No. (%)		
Hypothyroidism	Age (years)*	50.7 ± 7.786	45.3 ± 10.045	0.37
Age groups	20-29	8 (8.2)	4 (4.0)	
	30-39	20 (20.4)	6 (6.0)	
	40-49	26 (26.5)	21 (21.0)	
	50-59	43 (43.9)	67 (67.0)	
	≥ 60	1 (1.0)	2 (2.0)	
Hyperthyroidism	Age (years)	46.33 ± 11.855	45.3 ± 10.045	0.11
Age groups	20-29	7 (10.6)	4 (4.0)	
	30-39	12 (18.2)	6 (6.0)	
	40-49	15 (22.7)	21 (21.0)	
	50-59	24 (36.4)	67 (67.0)	
	≥ 60	8 (12.1)	2 (2.0)	

*Age was presented in years ± standard deviation.

**Table 2:** *MTHFR g.677C > T* and *MTHFR* A1298 genotypes and allele types and risk of thyroid disease

Condition	Genotype or allele	No. (%)*		Odds Ratio (95% CI)	*p*

		Cases	Controls		
Hypothyroidism	*MTHFR g.677C > T*				
N = 100	CC	49 (50.0)	55 (55.6)	1 (reference)	
	CT	32 (32.7)	32 (32.3)	0.63 (0.27-1.45)	0.28
	TT	17 (17.3)	12 (12.1)	0.71 (0.29-1.71)	0.44
	CC+CT	81 (82.7)	87 (87.9)	1 (reference)	
	TT	17 (17.3)	12 (12.1)	0.48 (0.20-1.14)	0.096
	C	130 (66.3)	142 (71.7)	1 (reference)	
	T	66 (33.7)	56 (28.3)	0.73 (0.44-1.21)	0.22
	*MTHFR g.1298A > C*				
	AA	45 (45.9)	37 (37.8)	1 (reference)	
	AC	37 (37.8)	29 (29.6)	2.43 (1.16-5.10)	0.019
	CC	16 (16.3)	32 (32.7)	2.55 (1.18-5.52)	0.017
	AA+AC	82 (83.7)	66 (67.3)	1 (reference)	
	CC	16 (16.3)	32 (32.7)	2.96 (1.44-6.11)	0.003
	A	127 (64.8)	103 (52.6)	1 (reference)	
	C	69 (35.2)	93 (47.4)	1.79 (1.07-2.99)	0.027
Hyperthyroidism	*MTHFR g.677C > T*				
N = 66	CC	51 (77.3)	55 (55.6)	1 (reference)	
	CT	10 (15.2)	32 (32.3)	2.94 (0.89-9.74)	0.078
	TT	5 (7.6)	12 (12.1)	1.05 (0.27-4.07)	0.95
	CC+CT	61 (92.4)	87 (87.9)	1 (reference)	
	TT	5 (7.6)	12 (12.1)	1.72 (0.57-5.52)	0.333
	C	112 (84.8)	142 (71.7)	1 (reference)	
	T	20 (15.2)	56 (28.3)	2.23 (1.12-4.45)	0.023
	*MTHFR g.1298A > C*				
	AA	6 (9.1)	37 (37.8)	1 (reference)	
	AC	46 (69.7)	29 (29.6)	0.42 (0.14-1.26)	0.123
	CC	14 (21.2)	32 (32.7)	4.01 (1.79-9.01)	0.001
	AA+AC	52 (78.8)	66 (67.3)	1 (reference)	
	CC	14 (21.2)	32 (32.7)	1.83 (0.88-3.79)	0.104
	A	58 (43.9)	103 (52.6)	1 (reference)	
	C	74 (56.1)	93 (47.4)	0.18 (0.07-0.48)	0.001

* Data represent actual numbers with percent in parentheses.

The haplotypes appearing in our results are CA, CC, TA, and TC. The most frequent haplotypes were CA (677C-1298A) (hypothyroidism cases: 51.5%; hyperthyroidism cases: 45.9%; controls: 35.4%) followed by CC (677C-1298C) (hypothyroidism cases: 14.4%; hyperthyroidism cases: 39.0%; controls: 36.3%). The rarest haplotype in hypothyroidism and hyperthyroidism cases was TA: 13.3% and 5.0%, respectively. Our results indicated that the two loci 677 and 1298 show linkage disequilibrium (LD) between cases (D’_hypo_ = 0.39, D’_hyper_ = 0.25) and fair LD in controls (D’ = 0.14). Carriers of the CC (677T-1298A) and TA (677T-1298A) haplotypes had significantly lower risks for hypothyroidism, whereas those with TC (677T-1298A) haplotypes had a higher likelihood of having hypothyroidism (Table 3).

**Table 3:** Haplotype frequencies of MTHFR among hypothyroidism patients, hyperthyroidism patients and controls

Condition	Haplotype	Cases	Controls	Odds Ratio	(95% CI)	*p*

		No. (%)*				
Hypothyroidism	677C-1298A	101 (51.5)	69 (35.4)	0.84	0.46-1.53	0.57
	677C-1298C	29 (14.4)	70 (36.3)	0.24	0.12-0.47	0
	677T-1298A	26 (13.3)	32 (16.7)	0.47	0.23-0.97	0.04
	677T-1298C	40 (20.4)	23 (11.7)	2.14	1.03-4.44	0.04
Hyperthyroidism	677C-1298A	61 (45.9)	69 (35.4)	0.88	0.45-1.73	0.72
	677C-1298C	51 (39.0)	70 (36.3)	1	0.51-1.95	1
	677T-1298A	7 (5.0)	32 (16.7)	1	0.47-2.13	1
	677T-1298C	13 (10.2)	23 (11.7)	1	0.51-1.95	1

* Counts reflect the number of chromosomes.

## DISCUSSION

Since thyroid dysfunction and *MTHFR* polymorphisms are common among Jordanians, investigating the genes and polymorphisms involved in the folate metabolic pathway may assist in determining patient susceptibility to thyroid diseases and may help in early detection and management of the disease, particularly in childbearing-age females.

Although many molecular and epidemiological studies have been conducted worldwide in the past few decades concerning the relationship between *MTHFR* gene polymorphisms and cancer (e.g., lung, breast, and other types of cancers), only a few studies have been conducted on the correlation between these polymorphisms and the dysfunction of thyroid glands (17,18). Among the Japanese population, no association of g.677C>T or g.1298A>C polymorphisms and AITD leading to hypothyroidism or hyperthyroidism was detected; additionally, the genotype and allele frequencies of g.677C>T and g.1298A>C did not inﬂuence the prognosis of AITD (25). A meta-analysis involving 9 studies showed no association of g.677C>T polymorphism with thyroid disease, but an association was detected with thyroid cancer (OR _T vs. C_ = 1.09, 95% CI 0.94–1.26, *p* = 0.25; OR _TT vs. CC_=1.04, 95% CI 0.75–1.42, *p* = 0.83; OR _TT vs. CC/CT_=1.13, 95% CI 0.86–1.50, *p* = 0.37; OR _TT/CT vs. CC_ = 1.22, 95% CI 0.88–1.68, *p* = 0.24) (20). Our study involved two common polymorphisms and only female subjects were recruited; the g.1298A>C polymorphism showed more significant results than the g.677C>T polymorphism in our sample.

A familial history of AITD is commonly present. Genome-wide linkage analysis was the first approach employed to screen the genome for the genetic contribution to AITD. However, linkage studies lacked success for almost all complex diseases, suggesting that it can succeed for monogenic diseases but not for complex diseases. Linkage analysis has not revealed novel susceptibility loci for AITD, but the genetic contribution to AITD may have several effects that can be summarized as follows: (i) There was not a large genetic effect discovered for AITD, indicating that disease susceptibility might be due to small effects of multiple genes; (ii) Differences between the genetic contribution in Asian and Caucasian subjects suggest that different sets of genes may contribute to disease susceptibility in different environments and races; (iii) Different combinations of genes may lead to similar clinical phenotypes; (iv) Epigenetic phenomena may have a dominant influence; and (v) More comprehensive screening utilizing the new techniques with greater understanding of the genome is needed. PTPN22, IL-2RA/CD25, CD40, and SCGB3A2 genes were shown to be associated with Graves’ disease (GD) using candidate gene studies (23,24,26-31). HLA-DR3, cytotoxic T-lymphocyte-associated protein 4 (CTLA-4), and the TSHR genes were shown to be major susceptibility genes for GD and Hashimoto’s thyroiditis (HT) using the candidate gene approach (32). As discussed earlier, polymorphisms in various genes have been associated with AITD. The influence of each gene on AITD development when assessed in a population appears weaker than expected from the data that show strong genetic susceptibility to AITD. Explanations of this discrepancy include gene-gene interaction and subset effects. Further studies should be performed to elucidate the mechanism by which *MTHFR* deficiency would affect thyroid cellular functions and consequently the development of AITD.

As discussed previously, genetic factors are important in the pathogenesis of autoimmune thyroiditis. The co-occurrence of HT and GD within one family suggests a common genetic basis for these diseases (33). In the whole-genome screening of families, siblings, and populations with AITD, a number of sites have been located for GD and HT susceptibility, but none of them have shown significant statistical values (34-36). This result has been true for other autoimmune diseases, not just for AITD. This can be explained by HLA subtypes; for example, not every patient with GD has the associated HLA-DR3 subtype or even the associated Arg74 in its binding pocket, irrespective of the HLA-DR subtype (37). As a result, the disease can occur even in the absence of the expected HLA association.

The etiology of HT and GD involves common pathways that activate the escape of tolerance of thyroid-reactive T-cells and their infiltration of thyroid tissue as well as unique pathways that use the thyroid-reactive T-cells to induce thyroid cell death (in HT) or stimulation (in GD). As a result, genetic susceptibility to HT and GD involves shared genes and unique ones. Our results have shown similar effects of g.1298A>C polymorphism on hypothyroidism and hyperthyroidism, implying an influence on the common pathways.

Hence, mechanistic studies may assist in understanding the molecular basis of MTHFR’s role in the pathogenesis of AITD. Future studies should also include larger sample sizes to confirm results and establish an association that allows us to include *MTHFR* polymorphisms in the screening and examination of susceptible individuals. Once a mechanistic and statistical relationship is established, clinicians may introduce preventive guidelines for dealing with susceptible patients, including supplementation with commercially available 5-methyltetrahydrofolate (MTHFR product) to replace the reduced MTHFR function and reduce the risk of AITD.

Both genetic and environmental risk factors contribute to the development of thyroid diseases. One mechanism by which environmental factors may promote AITD together with genetic factors is by changing the epigenetic control of gene expression. So far, little is known about these interactions in AITD. However, there has been wide confirmation of the role of X chromosome inactivation (XCI) (38,39). Patients with AITD showed more biased expression of a maternal or paternal X chromosome than normal individuals, supporting the hypothesis that the poorly expressed chromosome may become active in certain tissues (such as the thyroid) and begin to express new antigenic sequences not previously recognized by the immune system. However, this potential mechanism for enhanced susceptibility to AITD needs further investigation.

Non-genetic risk factors also contribute to the development of autoimmune thyroid disease. These factors include radiation exposure, both from nuclear fallout and medical radiation, and high iodine intake, in addition to several other environmental contaminants that influence the thyroid (40-42). Exposure to these factors increases the probability of thyroid dysfunction; this may explain the inability of the current study to find a highly significant association between *MTHFR* polymorphisms and thyroid disease in this sample of patients.

The distribution of g.677C>T *MTHFR* genetic polymorphism was not significantly different between Jordanian females with hypothyroidism or hyperthyroidism and the control subjects. Individual *MTHFR* genetic polymorphisms might not independently affect the susceptibility to thyroid disease. Adjacent SNPs often show a high correlation between genotypes, meaning that they are in LD and that the interaction of the SNPs within haplotypes might act as a major determinant of disease susceptibility in comparison with the single polymorphisms (43). The analysis of haplotypes was reported by many studies to be more powerful than single polymorphism analysis (43). In this study, LD between g.677C>T and g.1298A>C in the *MTHFR* gene among hypothyroidism cases was detected. Our results show a significant increase in TC haplotypes among hypothyroidism patients compared to controls, implying a combined effect of g.677C>T and g.1298A>C polymorphisms.

The limitations of this study include the relatively small sample size and the difficulty of matching cases and controls on several variables other than age and gender. Additionally, healthy recruits (controls) were not tested to confirm that they were free of thyroid disease at the time of recruitment, nor were follow-up tests performed later. Moreover, data regarding folate intake, folate serum level, and pregnancy and abortion history were not adequately collected or not measured at all. No reliable valid method was developed to assess patients’ dietary intake of folate; this may be significant, as flour in Jordan is fortified with iron and folic acid under the auspices of the Ministry of Health and the vast majority of Jordanians eat bread daily (Food Fortification Initiative, 2014) (44). Finally, the search for genes that increase susceptibility to thyroid autoimmune diseases has identified several candidates, but they only account for a small percentage of the current prevalence of these disorders. Hence, other genes that may be involved in the modulation of thyroid disease risk must be investigated. Recruiting only females in this study was consistent with their higher susceptibility to autoimmune diseases.

In conclusion, the findings of our study suggest that genetic variants of *MTHFR* at g.1298A>C and its haplotype analysis at 677 and 1298 may modulate the risk of thyroid disorders in Jordanian females. This study is the sole one that has examined the role of *MTHFR* (g.677C>T and g.1298A>C) genetic polymorphisms and their haplotypes in the modulation of thyroid disease in the Jordanian population.
